# Quadriceps autograft to treat Achilles Chronic tears: a simple surgical technique

**DOI:** 10.1186/s12891-016-0967-1

**Published:** 2016-03-05

**Authors:** Rafael Arriaza, Raquel Gayoso, Emilio López-Vidriero, Jesús Aizpurúa, Carlos Agrasar

**Affiliations:** Instituto Médico Arriaza y Asociados, Calle Enrique Mariñas, 32, 15008 La Coruña, Spain; International Sports Medicine Clinic, Calle Arjona, 10, 41001 Sevilla, Spain; Cátedra de Traumatología del Deporte HM, Universidade da Coruña, Avenida Che Guevara 121, Oleiros, 15179 A Coruña, Spain

**Keywords:** Chronic Achilles tendon rupture, Surgical reconstruction, Quadriceps autograft, Platelet rich plasma

## Abstract

**Background:**

Chronic Achilles tendon tears could hinder patients and represent a challenge to surgeons. Although many different surgical techniques have been proposed for reconstruction of a neglected Achilles tendon rupture, there is no clear evidence to support one technique over the others, but the use of a technique that could allow for an “anatomical” reconstructions seems desirable.

**Methods:**

The present paper describes a new anatomic Achilles tendon reconstruction for chronic tears, using a quadriceps tendon autograft as graft source, with PRP injected into the graft and the neighbor tissue, and fixation in a bone trough with a simple small fragments screw.

**Results:**

Autologous quadriceps tendon graft seems an excellent option, although -surprisingly- has received little attention until now.

**Conclusions:**

Autologous Quadriceps tendon graft (in bone-tendon configuration) is a simple technique that could allow surgeons to reconstruct tissue defects in the Achilles tendon with non-expensive hardware.

## Background

**Fig. 1:**
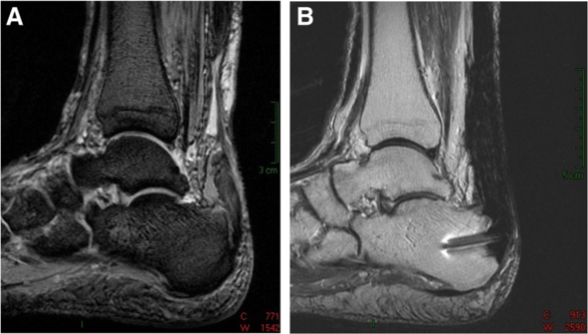
**a** and **b** MRI of a 72 year old patient, before surgery (9 months after Achilles tendon tear), and 8 months after surgery with autologous quadriceps tendon graft

Achilles tendon ruptures represent the most common acute tendon rupture in the human body, and frequently they are diagnosed solely based on clinical examination, but it is considered that up to 25 % of them can be misdiagnosed by the first clinician who examines the patient [1, 2]. Some of them can take more than 4–6 weeks to get correct diagnosis and treatment, and are considered as chronic, neglected or delayed in medical literature, and represent a challenging therapeutic problem due to the retraction and scarring of the tendon stumps, that create a gap that could be several centimeters long (Fig. 1a). Although there is no consensus regarding the specific time in which an acute tear becomes a chronic rupture, 4 weeks may be the most widely accepted interval [3, 4], though in fact, in some cases it is more important to note the resultant gap between the tendon stumps than the time elapsed since the rupture. Regardless of the lack of a precise chronological definition, neglected ruptures are characterized by the difficulty of achieving an end-to-end apposition of the tendon ends with plantar flexion of the foot during surgical reconstruction.

In the past, several authors [5–7] recommended the use of an inverted superficial turn-down flap of the gastrocnemius fascia to cover the defect. Many other authors have used different tissues to bridge the tendon gap, from local tendons, such as flexor hallucis longus [8] or peroneus brevis tendon [9], to distant tendons, such as the semitendinosus [10] or gracilis tendons [11]. Although the use of the quadriceps tendon for the treatment of chronic Achilles tendon ruptures was first published in 1995 [12], we have found only anecdotic further references to such technique, without publications in English literature except to treat distal Achilles tendinosis [13] and, in fact, no consideration to this option was included in the revision of the history of graft options for neglected Achilles tendon ruptures published by the Cleveland Clinic team in 2008 [14].

Although platelet rich plasma (PRP) has been used with success in the treatment of acute Achilles tendon ruptures [15], a literature search yielded no results on its use for the treatment of chronic Achilles tendon ruptures, but the authors have been using PRP regularly in the last 10 years for the treatment of tendon injuries. and, to our knowledge, this is the first report on the results obtained with the use of an autograft and PRP in this difficult condition.

The present paper describes a new and simple surgical technique to obtain an anatomic Achilles tendon reconstruction, using a quadriceps tendon autograft as graft source, with PRP injected into the graft and the neighbor tissue, and fixation in a bone trough with a simple small fragments screw.

## Surgical technique

### Preparation and patient positioning

The patient is positioned in lateral decubitus position, on the uninjured side, tilted ventrally. Either general or regional anesthesia could be used, and prophylactic antibiotic in injected before thigh tourniquet is inflated. The lower limb is prepped and draped free. By external hip rotation with both the hip and the knee flexed, the assistant keeps the limb in a position in which the that the patella is pointing to the ceiling.

### Quadriceps tendon graft harvesting

A 5 cm skin incision is usually enough to delineate and harvest the bone-tendon quadriceps graft, with the standard technique. Usually, a 1 cm wide graft, with a 12 mm long bone plug and a 8–9 cm tendon length is obtained. After skin closure, the patient is rotated to ventral decubitus, which can easily be done without changing drapes. In this position, the Achilles tendon to be operated is now pointing to the ceiling, and the surgeon can work in a comfortable position.

### Achilles tendon grafting

**Fig. 2:**
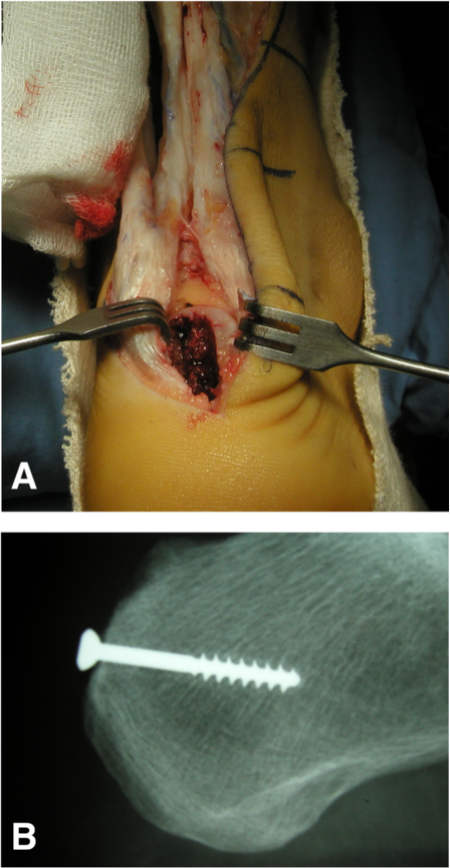
**a** and **b** Creation of a bone trough in the posterior aspect of the calcaneus, and fixation of the bone plug with a small fragment cancellous screw

**Fig. 3:**
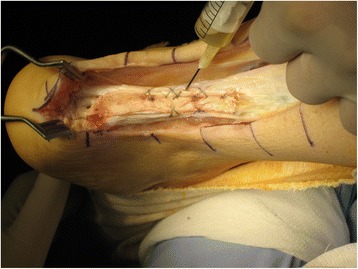
Injection of Leukocyte-Poor Platelet-Rich Plasma into the graft and the Achilles tendon stumps

A standard paramedial Achilles incision, extended distally over the calcaneus tuberosity, is used. Careful dissection of the sural nerve is performed, taking into consideration the possibility that scar tissue or edema could be found surrounding it. After necrotic and scarred tendon tissue are debrided, a trough is created in the posterior aspect of the calcaneus, to accommodate the bone plug from the patella, and the plug is fitted into the trough and secured with a 3.5 mm cancellous screw (Fig. 2a and b). Next, the proximal and distal tendon stumps are longitudinally divided, and the quadriceps graft is sutured latero-laterally to them, keeping a tension that places the foot in slight (approximatelly 15°) equinus. Leukocyte-Poor Platelet-Rich Plasma is injected into the graft and the Achilles tendon stumps, and the wound is closed (Fig. 3).

### Rehabilitation

After surgery, the patients are inmobilized with a below-knee cast for 3 weeks, and an orthesis with range of motion control is used thereafter, allowing free plantarward motion but limiting dorsiflexion to −20° for the next 2 weeks while allowing partial weight bearing. The orthesis is retired for night rest and physiotherapy, avoiding dorsiflexion above neutral until the 8th postoperative week.

## Discussion

The main finding of this study is that autologous quadriceps bone-tendon graft offers an excellent option for the repair of chronic Achilles tendon ruptures.

Quadriceps bone-tendon graft allows the repair of the Achilles tendon defect without any deleterious effect on neighbor structures, while offering an “anatomical” repair, as the bone plug can be placed in the tendon footprint at the posterior aspect of the calcaneus, avoiding modifications in the traction vector of the tendon. To the best of our knowledge, however, no surgical method using a quadriceps tendon graft fixed in a bone trough at the anatomical insertion site of the Achilles tendon at the calcaneus can currently be found in the literature. The reason for developing this new method was to bridge the tendon gap using a fairly simple procedure with a graft that is commonly used by sports surgeons, and to avoid the use of local tendons, as this option is not free of complications and possible functional imbalances [16].

Achilles tendon ruptures seem to have increased during the last two decades (in some areas, figures show a four-fold increase in incidence), probably due to the increase in the general population activity and sports participation [17]. A secondary consequence is an increase in the number of Achilles tendon ruptures not diagnosed properly during the initial medical visit, and also the increased number of patients with complications from the treatment -either surgical or conservative- employed to treat those ruptures. Those complications include re-ruptures, infections and tissue necrosis that could require further surgical procedures to correct the residual defect in the tendon, making it mandatory to use some form of graft tissue to bridge the defects [18]. Several surgical techniques have been proposed for reconstruction of a neglected Achilles tendon rupture, although there is no clear evidence to support one technique over the others, as the ultimate goal of the surgical treatment is to regain enough plantar flexion power in the ankle, and most techniques seem to reach this goal. In this sense, autologous quadriceps tendon graft seems an excellent option, although -surprisingly- has received little attention until now [12]. It is well known that quadriceps tendon autograft represents a reliable option for the treatment of several sports injuries, including ACL tears, and its use has seen a renewed interest, due in part to its excellent mechanical properties, the solid fixation that can be obtained using its bone plug if harvested as a bone-tendon unit, and the low morbidity associated with this procedure [19].

One of the most populat grafts for chronic Achilles tendon reconstruction is the FHL tendon, due to its proximity to the Achilles tendon, but its average lenght when harvested through a posterior incision is only 5.16 cm, which could prove insufficient to cover a higher defect [20], and for that reason, does not offer any special advantage to the use of the quadriceps tendon graft, which is usually longer.

After surgery, the foot is placed in equinus position to reduce the tension in the graft tissue and to increase skin vascularization, in order to minimize the risk of skin necrosis [21]. The use of PRP injection into the margins of the tendon graft and the host tissue was done to promote tissue healing, as some studies have shown an increase in the strength of the Achilles tendon in rats after PRP injection [22], and a faster recovery after PRP injection associated with surgical repair of the Achilles tendon in athletes [15]. Although, to our knowledge, this procedure has not been published in association with the surgical repair of chronic Achilles tendon tears, we opted to use the PRP injection of the repair site as the biologic environment created by the surgical procedure seems similar to the one of an ACL repair with an autologous graft tissue, in which the addition of PRP has been shown by some authors to promote an early integration and maturation of the graft [23]. We do acknowledge that there is still not enough scientific evidence to support the regular use of PRP in these surgical procedures, and a future randomized study might give very valuable information regarding its importance in the results obtained with the quadriceps tendon graft in cases of chronic Achilles tendon tears with marked retraction.

Limitations of this procedure lie basically in the distance of the tendon gap from the calcaneus: if is located more than 5–6 cm away from the calcaneus, the quadriceps graft might not have length enough to bridge it, in which case should opt for a different surgical procedures, as the ones recently described with a free tendon graft, as suggested by Sarzaeem [24] and Maffulli [25], a scar tissue repair and FHL augmentation, as proposed by Lee [26], or a free gastrocnemius aponeurosis flap, as proposed by Nilsson-Helander [10]. Also, this is a technical note, not presenting clinical and radiological follow-up data. In order to demonstrate the clinical significance and safety of this technique, clinical and radiological follow-up studies will be needed. A case series will be presented shortly when operated patients will have reached a significant follow-up.

## Conclusions

Autologous Quadriceps tendon graft (in bone-tendon configuration) is a simple technique that could allow surgeons to reconstruct tissue defects in the Achilles tendon. It could be used not only in cases of chronic or neglected Achilles tendon ruptures, but also in cases of surgical complications (as deep infections or Achilles tendon necrosis), without altering the function of the neighbor structures, nor shortening the myotendinous triceps surae unit.

## Consent

Written informed consent was obtained from the patient for the publication of this report and any accompanying images.

## Abbreviation

PRP: Platelet-rich plasma
